# Identification of fragment ions produced by the decomposition of tetramethyltin and the production of low-energy Sn^+^ ion beam

**DOI:** 10.1371/journal.pone.0253870

**Published:** 2021-06-25

**Authors:** Satoru Yoshimura, Satoshi Sugimoto, Takae Takeuchi, Kensuke Murai, Masato Kiuchi

**Affiliations:** 1 Center for Atomic and Molecular Technologies, Graduate School of Engineering, Osaka University, Suita, Osaka, Japan; 2 Faculty of Science, Department of Chemistry, Biology and Environmental Science, Nara Women’s University, Nara, Nara, Japan; 3 National Institute of Advanced Industrial Science and Technology (AIST), Ikeda, Osaka, Japan; Mohanlal Sukhadia University, INDIA

## Abstract

Tetramethyltin was decomposed in an ion source and the fragment ions produced were identified using a low-energy mass-selected ion beam machine. Dominant fragment ions were found to be H^+^, CH_2_^+^, and Sn^+^. Subsequently, fragment ions were mass-selected. The mass spectrum of the selected ions indicated that only a single peak appeared at the mass number of 120 u, being suggestive of the presence of ^120^Sn^+^ ions. The ion energy was set at the range of 20–100 eV. The Sn^+^ ion beam was irradiated to a Si substrate, and a film was then found deposited on the substrate after the ion beam irradiation. An X-ray diffraction measurement showed that the film obtained was metallic Sn. Then, the Sn^+^ ion beam was irradiated to a quartz crystal microbalance substrate. We found that most of the irradiated Sn^+^ ions were adhered to the substrate, at the ion energy levels of 25 and 58 eV, producing the Sn film, whereas a 107 eV Sn^+^ beam caused a significant proportion of Sn atoms in the film to detach from the substrate, probably due to sputtering.

## Introduction

Low-energy ion beam technique is recognized to be of use for various film-type material formations (e.g., iron [1], carbon [2], silicon carbide [3], and silicon dioxide [4]). On the other hand, the formation of films containing tin (Sn) atoms draw much attention because Sn-containing films (e.g.; tin oxide) are useful for many actual applications such as gas sensors [5], solar cells [6], and touch screens [7]. Those Sn-containing films can be fabricated by many experimental techniques such as chemical vapor deposition (CVD) [8], plasma-enhanced CVD [9], evaporation [10,11], sol-gel technique [12], and spray pyrolysis [13,14]. However, only a few experiments have been performed to form Sn-containing materials using Sn ion beams [15–17].

Meanwhile, tetramethyltin (TMT, (CH_3_)_4_Sn) seems to be an appropriate source material for Sn-containing film formations [18–28]. Fragmentation [19,29], pyrolysis [30], thermolysis [31], and plasmolysis [32] of TMT have been investigated.

We have investigated fragment ions produced from several different source materials such as methylsilane [33], dimethylsilane [34], hexamethyldisilane [35], hexamethyldisiloxane [36], tetraethylorthosilicate [37], hexamethyldisilazane [38], and hexamethyldigermane [39] in a Freeman-type ion source. Although the fragmentation of TMT has already been reported [19,29], no experiments have been performed to identify fragment ions produced from TMT in ion sources and to produce low-energy beams of Sn ions through the decomposition of TMT. In this study, fragment ions produced from TMT in a Freeman-type ion source were firstly identified. Low-energy Sn^+^ ion beams were then produced through the decomposition of TMT as a first step for the Sn-containing film formation experiments using the low-energy ion beam technique. For comparative purposes, Sn^+^ ion beams were also produced by the sputtering of tin oxide target. We tried to characterize the Sn^+^ ion beam obtained. Then, the Sn^+^ ion beam was irradiated to a Si substrate. The resulting film deposited on the substrate was examined by X-ray diffraction (XRD). Finally, the Sn^+^ ion beam was irradiated to a quartz crystal microbalance (QCM) substrate and the ion energy dependence of self-sputtering yields of Sn was measured. The measurement of the ion energy dependence of the self-sputtering yields is useful for determining both ion energy and substrate bias voltage in the ion beam deposition experiments.

## Materials and methods

**Fig 1:**
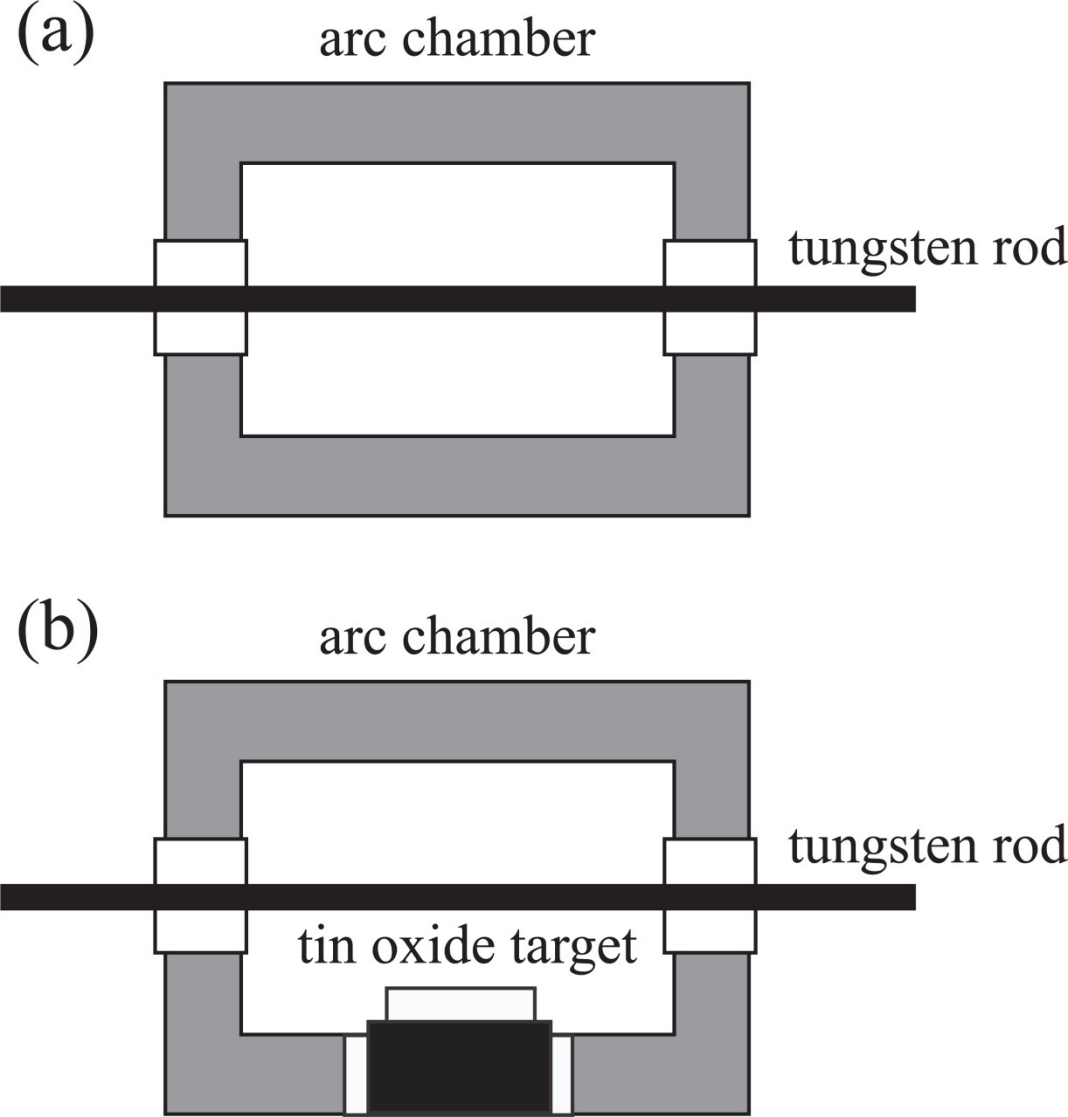
Schematic diagrams of (a) an ordinary Freeman-type ion source and (b) a modified Freeman-type ion source for a low-energy mass-selected ion beam machine.

Experiments were performed using a low-energy mass-selected ion beam machine (Ulvac). The schematic drawing of the machine was shown in our previous paper [40]. The machine is composed of a Freeman-type ion source, a mass selector, and a processing chamber. The ion source consists of an arc chamber and a hot tungsten rod, as shown in Fig 1A. The hot tungsten rod is used as an electron emitter.

We employed TMT as a source material for the Sn^+^ ion beam production. TMT was bubbled with Ar gas. The resulting mixed gas of TMT and Ar was led into the ion source at 2 sccm. TMT was decomposed by the electron impact in the ion source. In this study, Ar gas was used for the bubbling of TMT because we found that the mixed gas of TMT and Ar could be steadily ionized in the ion source.

A tin oxide (SnO_2_) sputtering target was also used as a source for the Sn^+^ ion beam production. In this case, we used a modified Freeman-type ion source. The schematic drawing of the modified ion source is shown in Fig 1B. The target was set inside the arc chamber of the ion source. The reason why we used SnO_2_ but not metallic Sn as a target is that the melting temperature of metallic Sn is too low to be placed inside the arc chamber. The melting temperatures of metallic Sn and SnO_2_ are 232 and 1630°C, respectively. For the Sn^+^ ion production using the SnO_2_ target, the arc chamber was firstly filled with Ar gas with a fixed gas flow rate (1 sccm). Thus, an Ar plasma was generated in the arc chamber and the Sn^+^ ions were sputtered from the SnO_2_ target by the Ar ion bombardment from the plasma.

Ions produced in both ion sources were extracted through the slit of the arc chamber by a high voltage of -15 kV. A mass selector was used to select Sn^+^ ions from several kinds of ions extracted from the ion sources. Then, the kinetic energy of the Sn^+^ ion beam was decelerated and the low-energy Sn^+^ ions were led into the processing chamber. The base pressure in the processing chamber was 1×10^−6^ Pa. In the processing chamber, the ion mass and ion energy were analyzed with a mass-energy analyzer (PPM-421, Balzers).

Although powdery SnO_2_ is cheap, solid SnO_2_ target to be set inside the ion source is expensive. Therefore, the experiments using the SnO_2_ sputtering target seem to be less profitable than experiments using TMT.

## Results

### Identification of fragment ions produced from tetramethyltin

**Fig 2:**
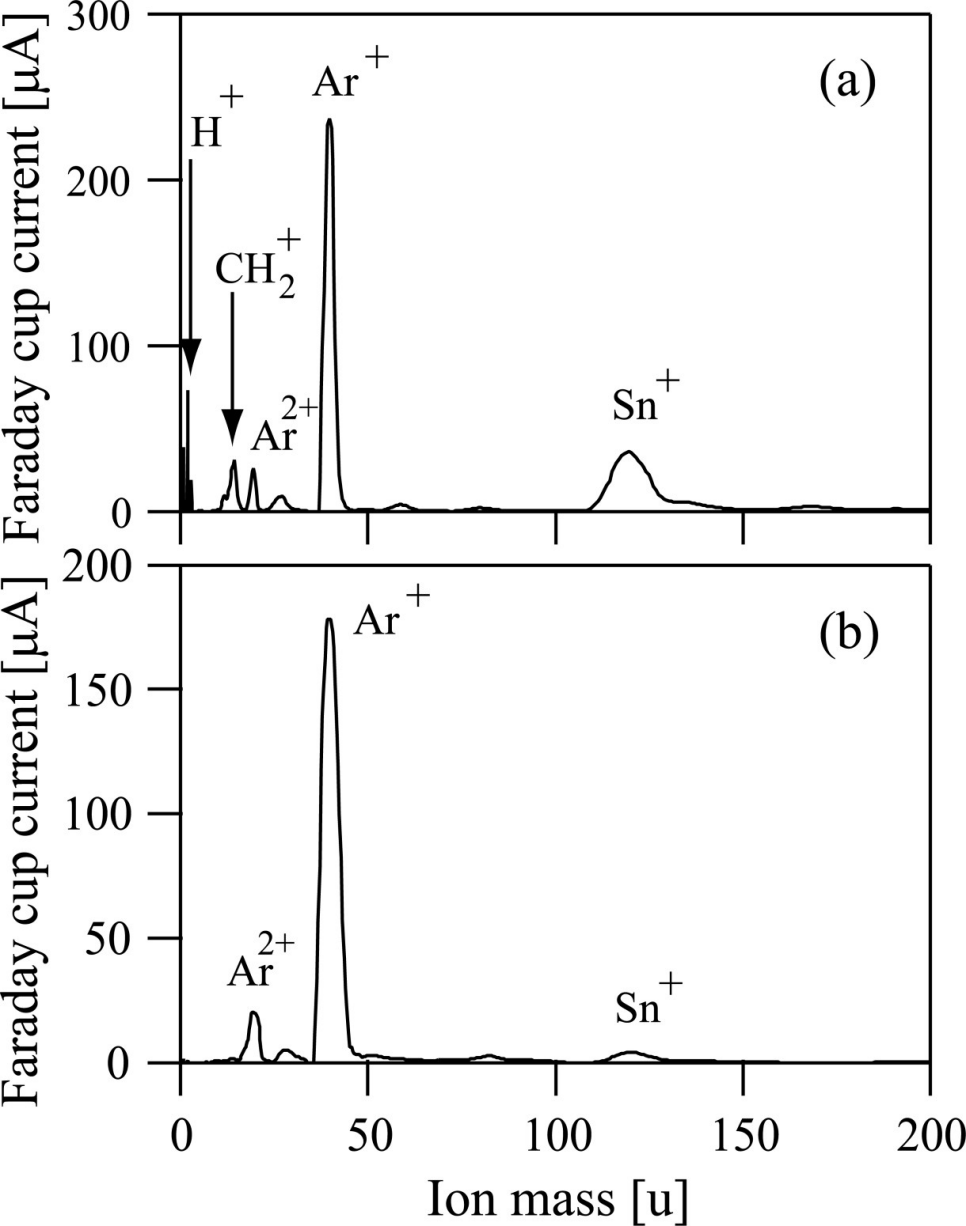
(a) The ion intensity measured by a Faraday cup for each ion species selected by a mass selector when tetramethyltin was used as a source material. (b) The ion intensity measured by a Faraday cup for each ion species selected by a mass selector when a tin oxide target was set inside the modified Freeman-type ion source.

A mixed gas of TMT and Ar was firstly introduced into the Freeman-type ion source and then TMT was decomposed to obtain fragments. Fragment ions can be detected by a Faraday cup set just behind the mass selector. Fig 2A shows a result of ion mass distribution measurements. In Fig 2A, three dominant peaks are observed in addition to peaks due to Ar^+^ and Ar^2+^. Fig 2A shows that dominant fragment ions produced from TMT are H^+^, CH_2_^+^, and Sn^+^. Fig 2A also shows that the beam current of Sn^+^ ions produced from the decomposition of TMT was about 40 μA.

Subsequently, ion mass distribution was also analyzed when an SnO_2_ target was set inside the modified Freeman-type ion source. The measured mass spectrum of ions revealed that Sn^+^ ions were presented in the ion source, as shown in Fig 2B.

### Production of low-energy Sn^+^ ion beam

**Fig 3:**
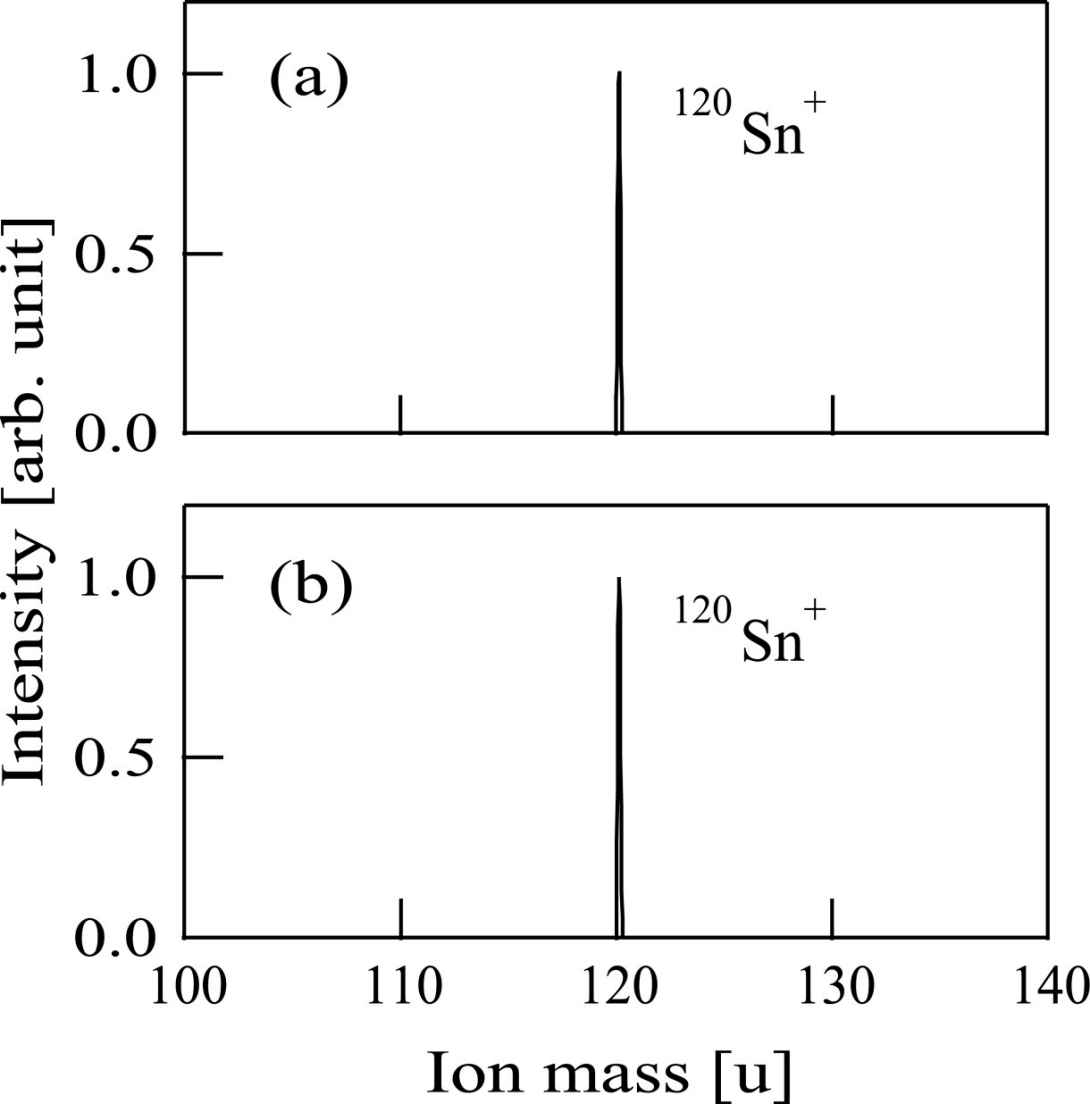
(a) The mass spectrum of selected fragment ions produced from tetramethyltin. (b) The mass spectrum of selected ions produced when a tin oxide target was set inside the modified Freeman-type ion source.

We can select desired ions using a mass selector. After the mass selection, the ion beam was decelerated and then guided into the processing chamber. In the processing chamber, the ion mass was analyzed with the mass-energy analyzer. The mass spectrum of selected ions, produced from TMT, is shown in Fig 3A. On the other hand, the spectrum of mass-selected ions obtained by the sputtering of the SnO_2_ target is shown in Fig 3B. Both Fig 3A and 3B show that only a single peak appeared at the mass number of 120 u, being suggestive of the presence of ^120^Sn^+^ ions. Although tin has many isotopes such as ^116^Sn, ^118^Sn, and ^120^Sn, we can select only ^120^Sn^+^ ions using this technique. We decided to choice ^120^Sn^+^ ions because ^120^Sn is naturally more abundant than the other Sn isotopes.

**Fig 4:**
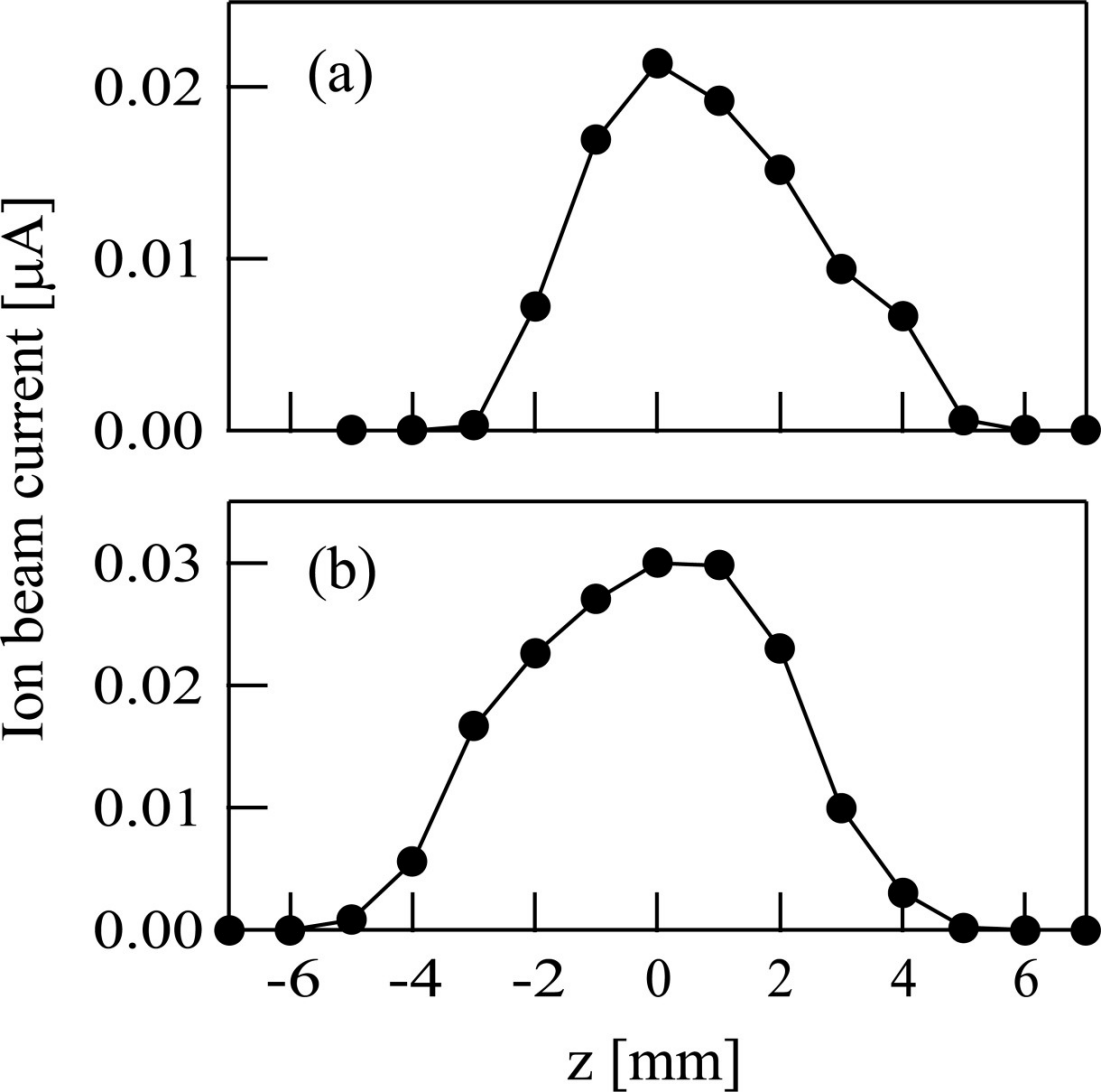
(a) The intensity profile of the Sn^+^ ion beam produced from tetramethyltin. (b) The intensity profile of the Sn^+^ ion beam when a tin oxide target was set inside the modified Freeman-type ion source. The horizontal axis represents the distance in the vertical direction in both figures (a) and (b).

The radial profile for the Sn^+^ ion beam was measured with a 2 mm orifice plate set in the processing chamber. The ion energy was set at 100 eV. The profile for the Sn^+^ ion beam produced from TMT is shown in Fig 4A. On the other hand, the profile for the Sn^+^ ion beam obtained by the sputtering of the SnO_2_ target is shown in Fig 4B. The full widths at half maximum (FWHMs) of ion beam profiles were 5 and 6 mm in Fig 4A and 4B, respectively.

**Fig 5:**
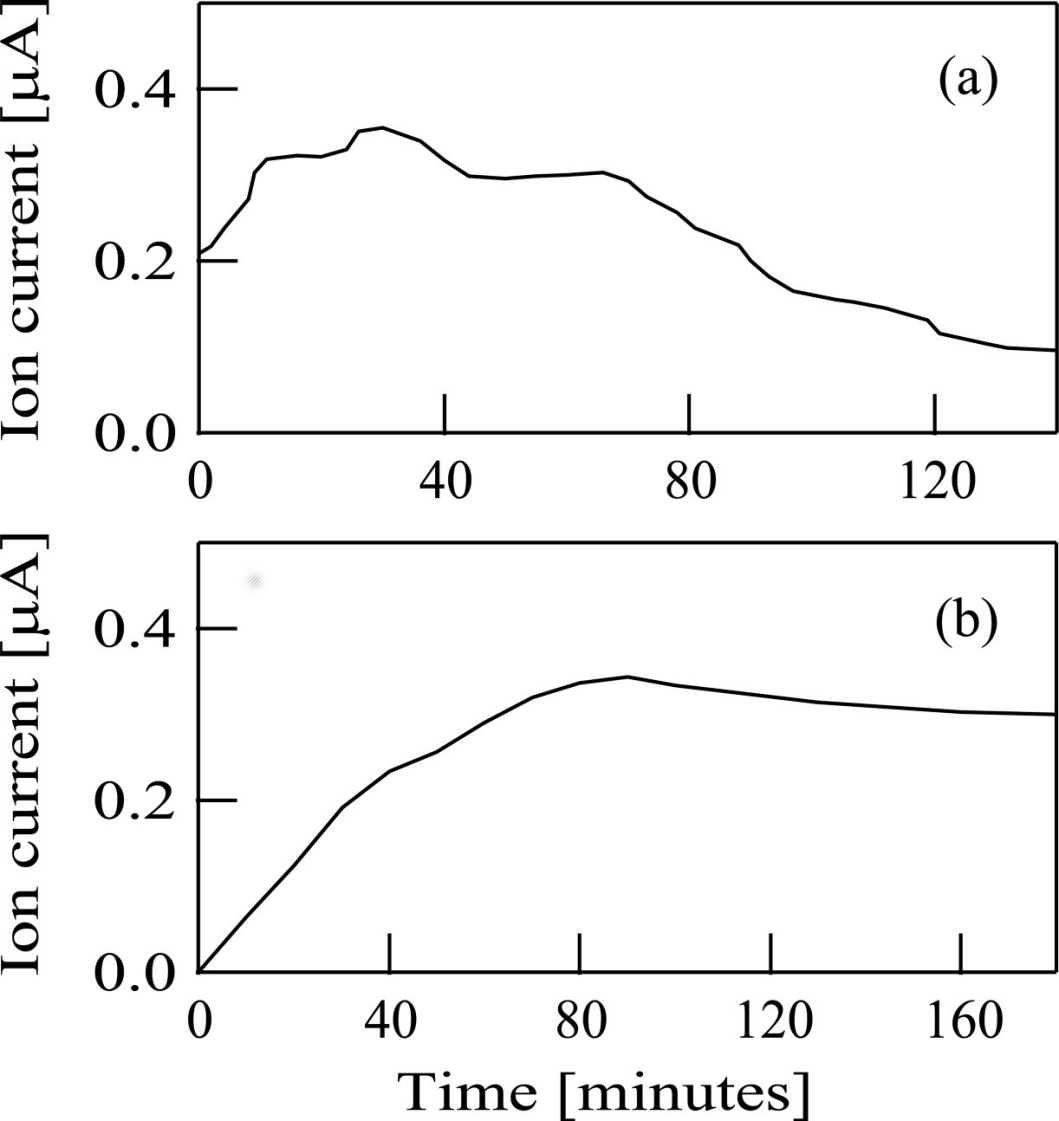
(a) The typical time course evolution of the Sn^+^ ion current when tetramethyltin was used as the source material. (b) The typical time course evolution of the Sn^+^ ion current when a tin oxide target was set inside the modified Freeman-type ion source. The vertical and horizontal axes in both figures (a and b) represent the ion current and the duration of the experiment respectively.

The time variations of the Sn^+^ ion beam currents were measured with a Faraday cup set in the processing chamber. The temporal evolution of the mass-selected ^120^Sn^+^ ion beam produced from TMT is shown in Fig 5A. The ion current increased gradually after the start of the experiment and a peak value reached at about 30 minutes after the start of the experiment. Then, the ion current decreased gradually. On the other hand, the temporal evolvement of the ^120^Sn^+^ ion beam obtained by the sputtering of the SnO_2_ target is shown in Fig 5B. The ion current increased gradually until about 80 minutes later and then the ion current intensity became almost constant, as shown in Fig 5B. After the measurements of the temporal ion beam evolution, we investigated conditions of the arc chamber of the ion source. We found that a thick film was formed on the inner wall of the arc chamber when the ^120^Sn^+^ ion beam was produced from TMT, suggesting that fragments produced from TMT deposited on the inner wall of the arc chamber. On the other hand, when the ^120^Sn^+^ ion beam was obtained by the sputtering of the SnO_2_ target, such film formations on the inner wall of the arc chamber were not observed. These results seem to be associated with the difference in the time course evolution of the Sn^+^ ion beams between Fig 5A and 5B.

### Irradiation of Sn^+^ ion beams to a Si substrate

**Fig 6:**
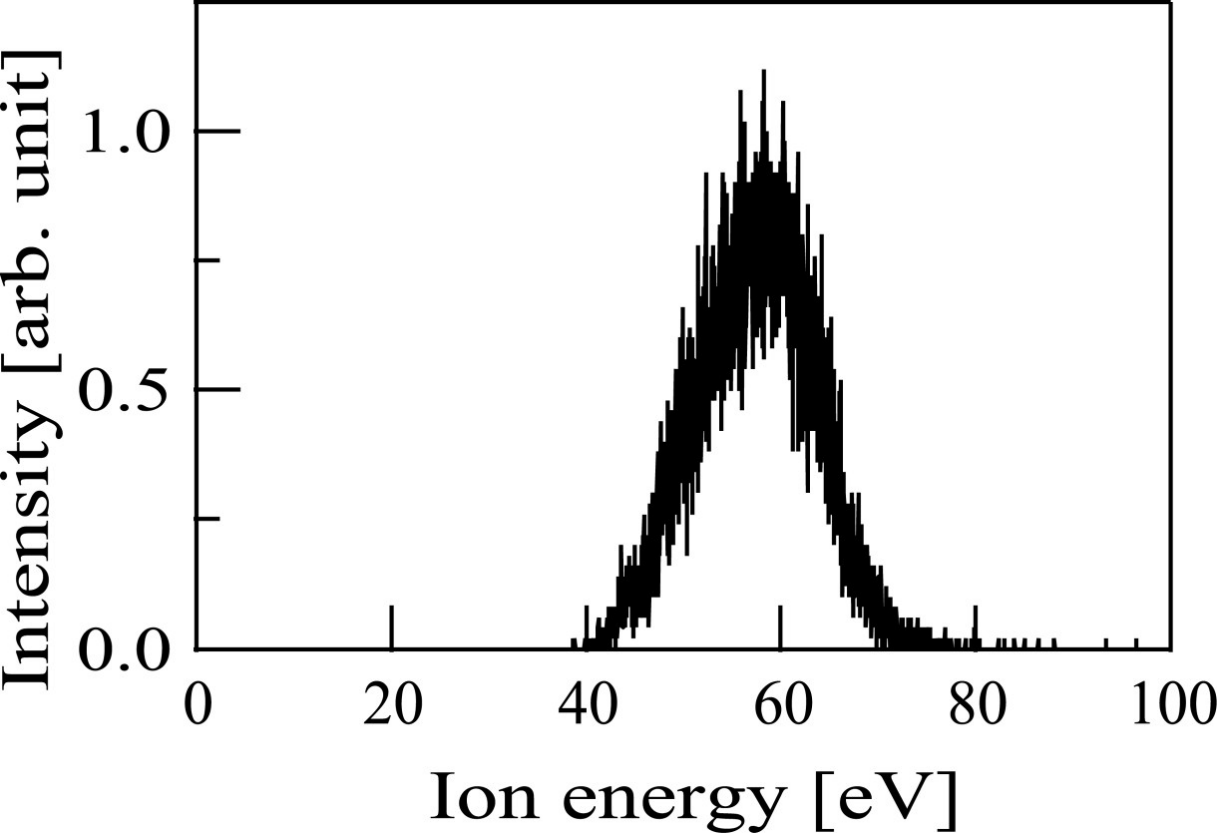
Energy distribution of the Sn^+^ ion beams produced when tetramethyltin was used as the source material.

Subsequently, the Sn^+^ ion beams were irradiated to a substrate. In this case, ^120^Sn^+^ ions were produced from TMT. Before ion beam irradiation, the energy distribution of the ion beams was measured by the mass-energy analyzer (Fig 6). Fig 6 shows that the peak energy is located at 58 eV and the FWHM of the spectrum is 13 eV. An untreated Si(111) substrate held at room temperature was used as a substrate. Average irradiated Sn^+^ ion current, *I*, was about 0.3 μA. The total time span of ion beam irradiation, *t*, was about 15 h. The total Sn^+^ ion dose irradiated to the substrate can be estimated by the equation, *It*/e, where e is the elementary electric charge. The estimated total Sn^+^ ion dose irradiated to the substrate was about 1 × 10^17^. Following the ion beam irradiation, the deposition of a film was found on the substrate. The film thickness (200 nm) was measured by a stylus profiler (P-15, KLA-Tencor).

**Fig 7:**
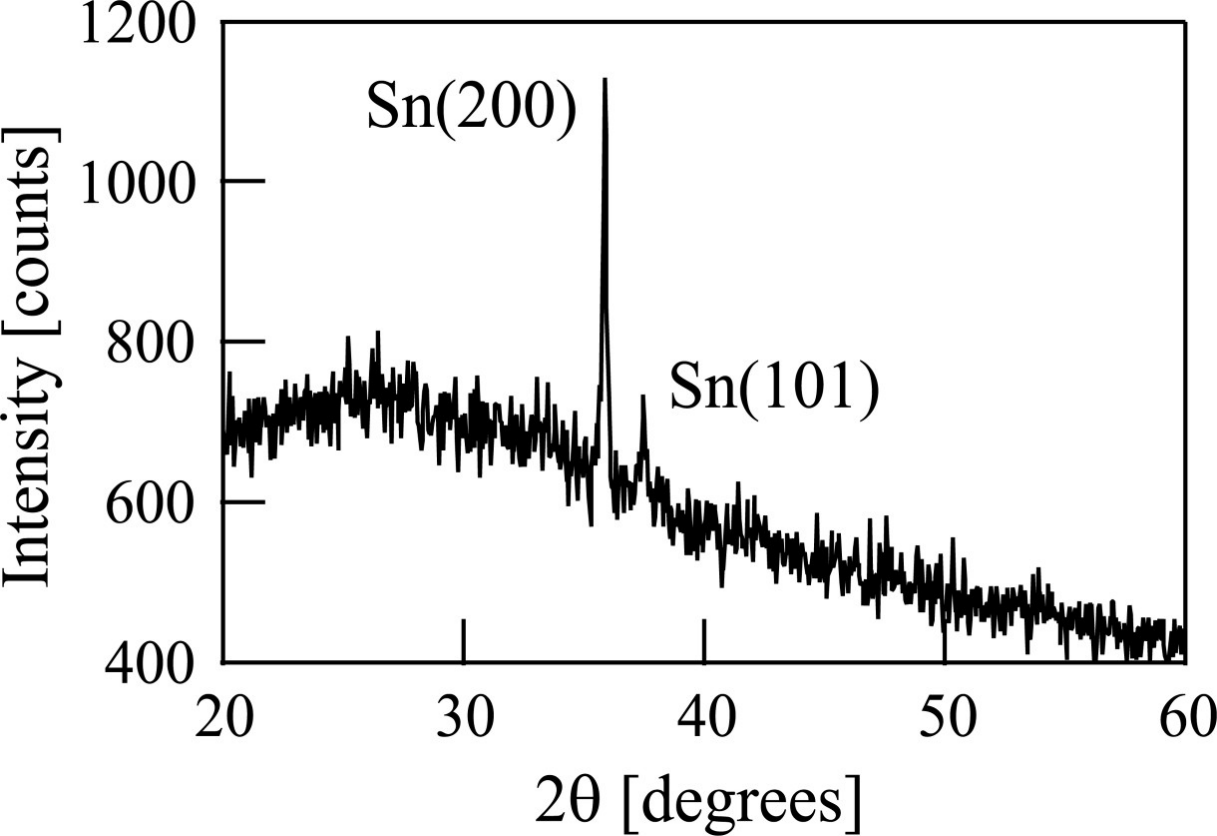
X-ray diffraction pattern of the film deposited on a Si substrate following the irradiation of Sn^+^ ions produced from tetramethyltin.

We then analyzed the film with XRD using an X-ray diffractometer (RINT2200, RIGAKU). The XRD pattern (θ-2θ method) obtained using k_α1_ of Co (1.78892 Å) is shown in Fig 7. Two peaks are observed in Fig 7. The Joint Committee on Powder Diffraction Standards (JCPDS) data (card number 40673) shows that these two peaks correspond to metallic Sn. The grain size of Sn was estimated to be 60 nm using the Scherrer formula [41].

### Irradiation of Sn^+^ ion beams to a quartz crystal microbalance substrate

**Fig 8:**
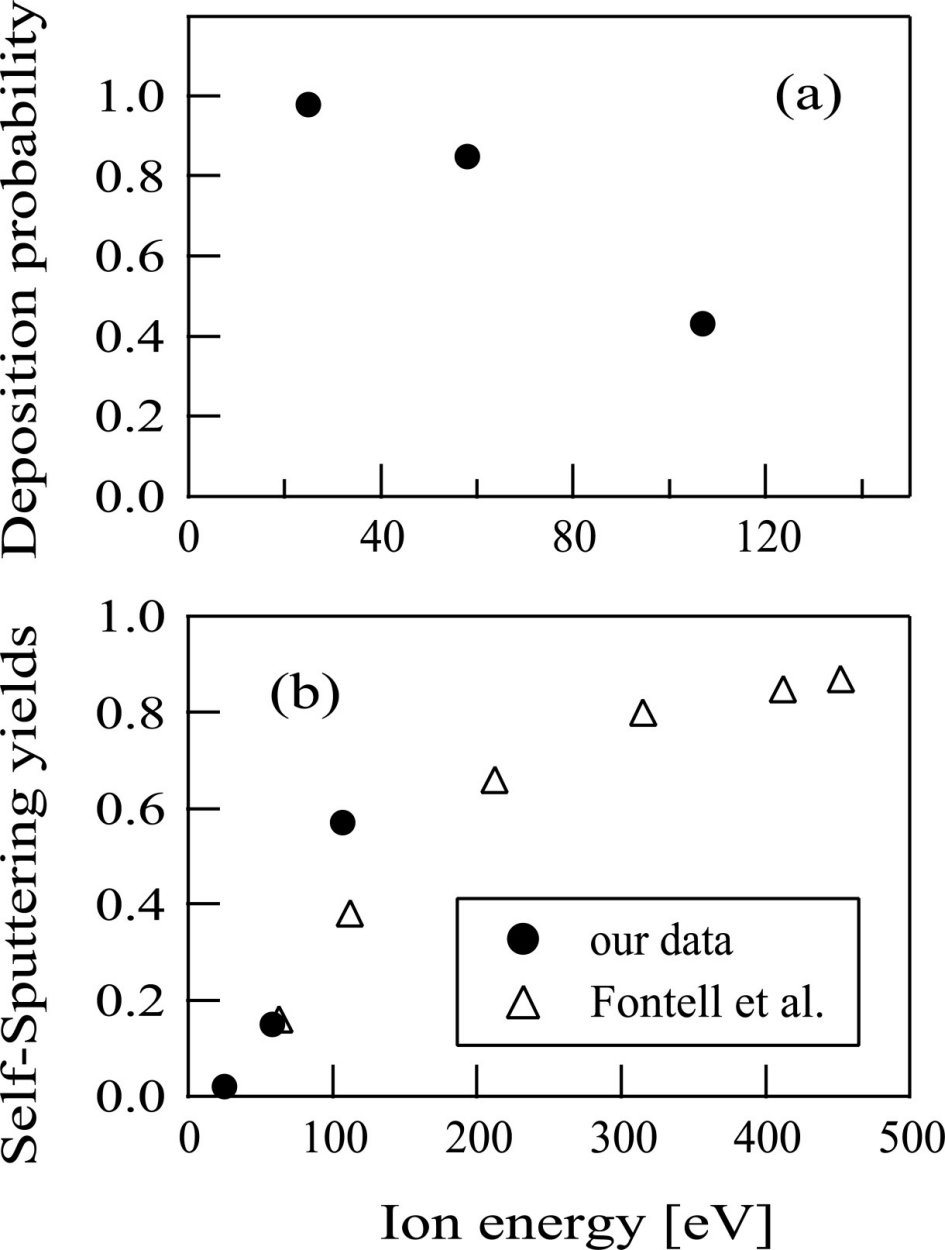
The measured deposition probabilities of Sn^+^ ions versus the ion injection energy are shown by closed circles in the figure (a). The self-sputtering yields of Sn are plotted by closed circles in the figure (b). In the figure (b), previous data presented by Fontell *et al*. [42] are also shown by open triangles for comparison.

A QCM substrate (CRTS-0, Ulvac) held at room temperature was used in this experiment as a substrate for the Sn^+^ ion irradiation. The substrate was an AT-cut crystal whose surface was coated with Au and whose resonant frequency was 5 MHz. Prior to Sn^+^ ion beam injection, a thin Sn film was formed on the QCM substrate surface by the Sn^+^ ion irradiation. We evaluated the mass increase of the deposited Sn film formed on the QCM substrate at the respective ion energies of Sn^+^ at 25, 58, and 107 eV using a QCM controller (CRTM-9000, Ulvac). The probability of irradiated Sn^+^ ions deposited on the substrate (here, we designate this value as “deposition probability” of Sn^+^ ions) was evaluated by calculating the ratio of the number of deposited Sn atoms on the substrate to that of incident Sn^+^ ions. The number of deposited Sn atoms on the substrate could be evaluated from the mass increase of the Sn film using CRTM-9000, whereas that of irradiated Sn^+^ ions could be calculated from the Sn^+^ ion current detected by a Faraday cup in the processing chamber before the Sn^+^ ion injection to the QCM substrate. Fig 8A shows the dependence of the deposition probability values on the Sn^+^ ion energy injected, and indicates also that more than 80% of the Sn^+^ ions were deposited on the substrate at both 25 and 58 eV. On the contrary, the deposition probability obtained using 107 eV Sn^+^ ions was only about 40%. It seems likely therefore that Sn atoms were ejected from the deposited film due to the sputtering effect, i.e., the film on the substrate was continually impacted by the 107 eV Sn^+^ ion beam.

The self-sputtering yields of Sn can be deduced from the deposition probabilities. The measured self-sputtering yields of Sn are plotted by closed circles in Fig 8B. In Fig 8B, the self-sputtering yields of Sn at ion energies of 60–450 eV presented in Ref. [42] are also plotted by open triangles. Fig 8B shows that the measured self-sputtering yield at the ion energy of 58 eV almost agrees with that in Ref. [42], whereas measured self-sputtering yield at ion energy of 107 eV was slightly larger than that in Ref. [42].

## Discussion

Several publications are available concerning the production of Sn ion beams [15–17,43]. Previous papers elucidated that Sn ion beams were useful for the doping of Sn atoms to semiconductors [15,16] as well as for the synthesis of Sn-containing alloys [17]. In those previous papers [15–17,43], source materials for Sn ion beams were metallic Sn. The authors of those papers [15–17,43] installed additional heating systems on their ion beam machines. In those experiments, the metallic Sn was heated to produce gaseous Sn atoms for the introduction of the Sn atoms into the ion sources.

Although TMT has attracted much attention as a source material for Sn-containing film formations [18–28], no experiments has been conducted to use TMT for Sn ion beam productions. In this study, we demonstrated that TMT was useful as a source material for Sn ion beam productions without installing an additional heating system on the ion beam machine.

The present study was attempted to determine whether Sn^+^ ion beams can be generated from TMT. Sn^+^ ion beam current obtained in this study was quite small and only 0.3 μA. It would be desirable for producing Sn containing materials with the ion beam techniques that the ion beam current of Sn^+^ ions is as great as possible. As shown in Fig 2A, the beam current of Sn^+^ ions just after mass-selected from TMT was approximately 40 μA. For the purpose of obtaining the higher mass-selection ability, a small slit was provided in the present ion beam machine and ion beams went through this small slit and then irradiated to the substrate set in the processing chamber. Owing to the presence of this slit, ^120^Sn^+^ ions could be isolated from among other various Sn isotopes. If the slit was removed from the ion beam machine, the Sn^+^ ion beam current would be significantly increased. However, the ion beams to be harvested will involve not only ^120^Sn^+^ ions but also other various Sn isotope ions (e.g., ^112^Sn^+^, ^114^Sn^+^, ^115^Sn^+ 116^Sn^+^, ^117^Sn^+^, ^118^Sn^+^, ^119^Sn^+^, ^122^Sn^+^, and ^124^Sn^+^). In addition to the slit removal from the ion beam machine, if we use other ion sources (e.g., Bernas-type) having ability to generate a higher ion beam, instead of Freeman-type one, the strength of Sn^+^ ion beam can be further increased. If the ion beam machine was remodeled as mentioned above, it is conceivable that the ion beam production of the mass-selected Sn^+^ ions from TMT can be employed for producing Sn-containing materials.

## Conclusions

In this study, fragment ions produced from TMT in a Freeman-type ion source were firstly identified. Dominant fragment ions were H^+^, CH_2_^+^, and Sn^+^. Sn^+^ ions were mass-selected and irradiated to a Si substrate. The temperature of substrate was set at room temperature. Following ion irradiation experiment, we discovered a clear film deposition on the substrate surface. XRD measurement indicated that the film obtained was metallic Sn. Then, the Sn^+^ ion beam was irradiated to a QCM substrate and the ion energy dependence of self-sputtering yields of Sn was measured. The measurements of the deposition probabilities of Sn^+^ ions showed that ion energy levels of 25 and 58 eV allowed most of irradiated Sn^+^ ions to deposit on the substrate. On the other hand, in the case of 107 eV a significant proportion of the injected Sn^+^ ions detached from the substrate, probably due to sputtering. Although the measured self-sputtering yield at the ion energy of 58 eV agrees with that in a previous paper [42], the self-sputtering yield at ion energy of 107 eV was slightly larger than that reported in the previous paper [42].

In conventional experiments for the Sn ion beam production, it was necessary to install a heating system for the production of gaseous Sn atoms on the ion beam machine. In this study, we demonstrated that TMT was useful as a source material for Sn ion beam productions without installing a heating system on the ion beam machine.

In this study, low-energy Sn^+^ ion beams were produced as a first step for the Sn-containing film formation experiments using the low-energy ion beam technique. Now, we have an experimental plan to form tin oxide films on substrates using the low-energy Sn^+^ ion beam in conjunction with an oxygen plasma irradiation.
